# Morphometric traits and identification of GH and IGF-1 gene polymorphisms in Napu buffalo (*Bubalus bubalis*) from Central Sulawesi, Indonesia

**DOI:** 10.5455/javar.2025.l887

**Published:** 2025-03-25

**Authors:** Amirudin Dg Malewa, Rusdin Rusdin, Mardiah Mangun, Isyana Khaerunnisa, Dwi Lestari, Yulius Duma

**Affiliations:** 1 Department of Animal Husbandry, Faculty of Animal Science and Fisheries, Tadulako University, Palu 94119, Indonesia; 2 Research Center for Applied Zoology, National Research and Innovation Agency, Bogor 16911, Indonesia

**Keywords:** IGF-1, GH, morphometric, Napu buffalo, polymorphism

## Abstract

**Objective:**

The objectives of this study are to measure the body weight (BW) and morphometric parameters of Napu buffaloes, identify the growth hormone (GH) and insulin-like growth factor-1 (IGF-1) gene polymorphisms, and associate them with the BW and morphometric parameters of Napu buffaloes.

**Materials and Methods:**

This study used 39 Napu buffaloes (9 males and 30 females). Morphometric measurements were performed using a measuring tape. The GH and IGF-1 gene diversity analysis was performed using polymerase chain reaction-restriction fragment length polymorphism (PCR-RFLP) and direct sequencing techniques.

**Results:**

The results showed that there is diversity in BW and body size of Napu buffaloes due to the influence of age and sex. The results of PCR-RFLP analysis of the GH|*AluI* gene showed 0% VV, 100% LL, and 0% VL genotypes. While the IGF-1|*SnaBI* gene shows the genotype AA 0%, BB 100%, and AB 0%. The sequencing results of the GH and IGF-1 genes in Napu buffaloes did not find mutations.

**Conclusion:**

Age and sex in Napu buffaloes may affect BW and body size. The GH and IGF-1 genes in Napu buffaloes are monomorphic, so no association can be made with the morphometric parameters of Napu buffaloes.

## Introduction

Buffalo is one of the ruminants used as a source of livelihood for Indonesian society, especially in rural areas, and has the potential to fulfill national meat needs. Buffaloes can utilize low-grade feed and adapt to marginal environments. Buffalo populations are spread across various regions in Indonesia, and almost 100% are mud buffalo/swamp buffalo, while river buffalo is less than 1% and only developed in North Sumatra [1].

Napu buffalo is a type of mud buffalo found in Central Sulawesi. Napu buffaloes are usually reared with traditional systems in pastures and rice fields. Napu buffaloes are usually used as labor to cultivate rice fields and as part of local customs [2].

According to BPS data [3], the buffalo population continued to decline from 2015 to 2022. The decline reached 15% and occurred in almost all regions of Indonesia, including the buffalo development base area, Sulawesi Province. The buffalo population in Sulawesi Province in 2015 amounted to 3,723 heads and decreased to 2,244 heads in 2022 [3]. The decline in buffalo population is caused by the active slaughtering of buffaloes and the trade of superior male buffaloes above the age of 4 years so that no one mates with females in heat. Uncontrolled population decline is feared to result in the extinction of the buffalo population. This will result in the loss of important genes related to livability and reproductive ability as well as genes that control economic traits. Therefore, efforts need to be made to anticipate future buffalo population declines.

Efforts that can be made to anticipate the decline in the buffalo population include maintenance management, reproduction management, and enhancing the livestock’s quality. Improving the genetic quality of livestock can be done by selecting economically valuable traits. One of the traits with high economic value is growth traits. Efforts to select for growth traits can be made based on buffalo body size (morphometrics). The body measurements of livestock can be utilized to estimate body weight (BW) and determine the selling price of livestock [4]. However, selection efforts based on body size will result in a slow selection response due to high environmental variation.

Selection programs using molecular markers (marker-assisted selection) as a tool have been carried out many times before. One of the gene groups that are widely utilized in the selection of growth traits is the growth gene group. Some of the growth gene groups include growth hormone (GH) and insulin-like growth factor-1 (IGF-1). The GH gene is responsible for encoding the GH hormone in animals. This hormone is a protein hormone that is produced and released by the anterior hypophyseal gland. GH is required for tissue growth, fat metabolism, and normal body growth [5]. Research by Hartatik et al. [6] and Pal and Chakravarty [7] stated that the GH gene is polymorphic and associated with growth traits. However, Soewandi et al. [8] found no association between GH and growth traits.

The IGF-1 gene, alongside the GH gene, has a significant impact on growth characteristics. The IGF-1 gene plays a role in various physiological and metabolic processes. The IGF-1 gene is a major candidate for traits of high economic value [9]. Ramesha et al. [10] found 7 single nucleotide polymorphism (SNP) of the IGF-1 gene in Indian cattle and buffaloes. In contrast, Putra et al. [11] stated that the IGF-1 gene in Pasundan cattle is monomorphic. Several studies found that the IGF-1 gene is associated with growth traits in Santa Ines sheep [12], milk yield [13], and carcass characteristics in New Zealand Romney sheep [14].

To the best of our knowledge, the available information on Napu buffalo is primarily limited to studies on female reproduction, including the estrus cycle, gestation duration, and calving interval [2], as well as general reproductive performance indicators such as birth rates, mortality, and population structure [15]. However, there is a notable lack of data on growth characteristics, particularly genetic markers associated with growth in Napu buffalo. Many studies have been conducted on GH and IGF-1 genes in cattle, chickens, and pigs. Research related to the diversity and association of GH and IGH-1 genes in Napu buffaloes has never been reported. Therefore, this study can provide basic information related to the diversity and association of GH and IGF-1 genes with BW and body measurements in Napu buffaloes. This study aims to measure the morphometric characteristics, identify the diversity of GH and IGF-1 genes, and associate them with the BW and body size of Napu buffaloes.

## Materials and Methods

### Ethical approval

This study was conducted in Lore Lindu National Park, Lindu District, Sigi Regency, Central Sulawesi. The animals used were 39 Napu buffaloes consisting of 9 males and 30 females aged I_0_ to I_1_. The experimental procedure was approved by the Animal Ethics Committee of the Faculty of Animal Science and Fisheries, Tadulako University (approval no. 05b/UN28.1.31/PT/2017).

### Tools and materials

The tools used for weighing buffaloes were ICONIX FX-1 (ICONIX, New Zealand) scales with a capacity of 200 kg, measuring sticks, measuring tape, cameras, and stationery. Tools used for blood sampling were BD VACUTAINER^®^ ECLIPSETM BLOOD COLLECTION NEEDLE, 21G X 1.25 (BD, US), and vacutainer tube.

### Data collection

**Figure 1.**
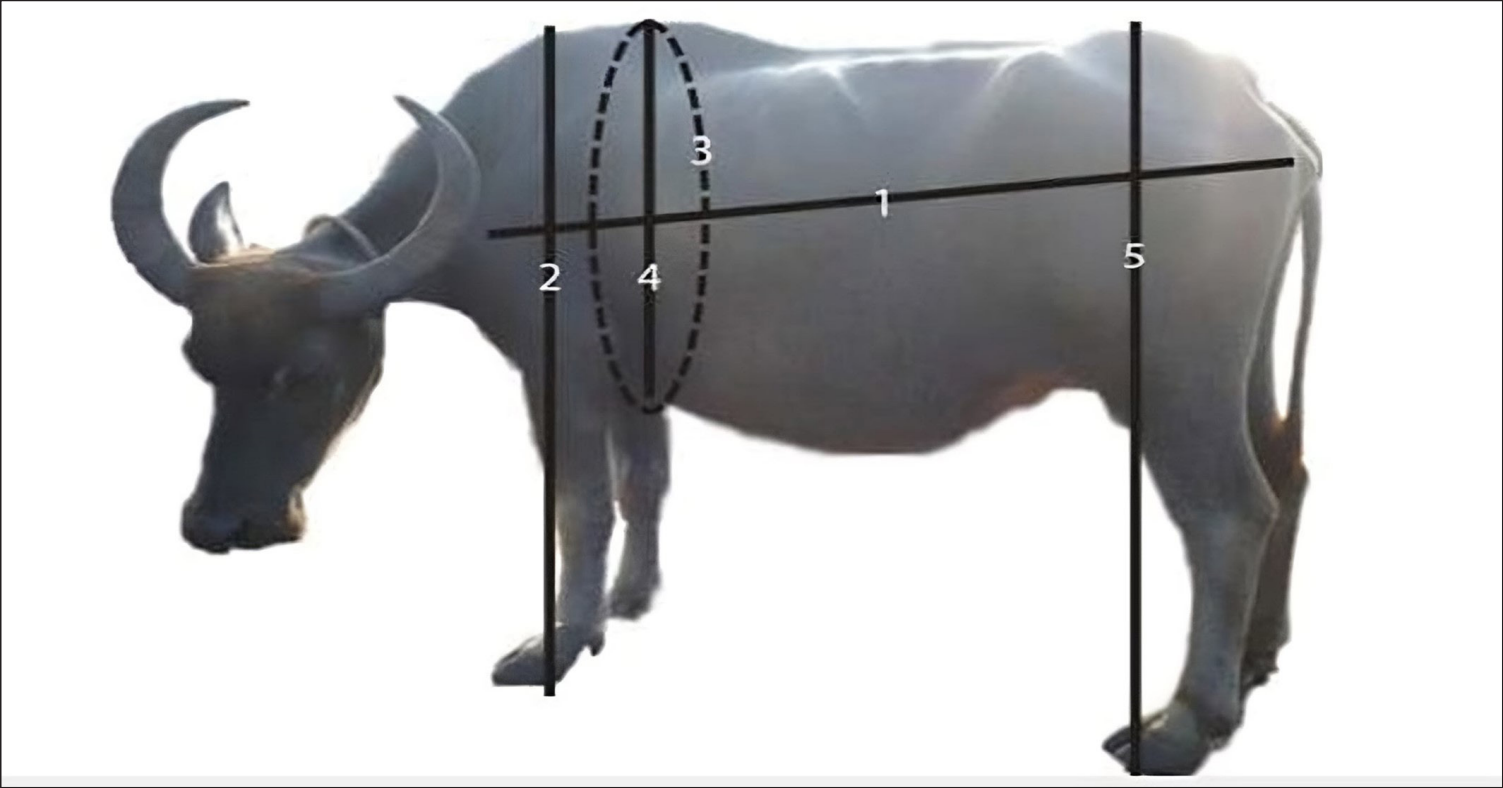
Measurements of buffalo body parts measured (in cm).

The survey approach is the one utilized to collect data. In the communities of the Lindu sub-district with the highest buffalo population, samples were taken using the purposive sample technique. The variables observed in this study were shoulder height (SH), body length (BL), chest girth (CG), chest depth (DD), hip height (HH), and BW. Measurements of buffalo body parts measured (in cm) and their definitions are described below (Fig. 1): (1) BL is the linear distance between the border of the precessus spinocus bone and the bump of the tapis bone (os ischium), measured using a yardstick in cm; (2) SH is the maximum vertical distance from the shoulder to the back of the scapula, measured perpendicular to the ground using a yardstick in cm; (3) CG is determined by measuring the circumference directly behind the scapula using a measuring tape in cm; (4) The chest depth (DD) is the vertical distance from the highest point of the shoulder to the sternum, which is measured with a measuring tape in cm; (5) HH refers to the maximum vertical distance from the hip to the ground, which is measured with a measuring stick in cm; and (6) BW, measured using a weighing scale in kg.

### Blood sampling

Blood sampling was conducted before the measurement of BW and body size. Blood sampling was done at 7–9 a.m. Blood samples were collected through the jugular vein in the neck with a 6 ml vacutainer needle and then put into a vacutainer tube containing 10% EDTA. Blood samples were then stored at 4°C until deoxyribonucleic acid (DNA) isolation.

### DNA isolation

The phenol-chloroform technique was performed to extract DNA. A 1.5 ml tube was filled with a blood sample (up to 100 µl) and 1,000 µl of 0.2% NaCl. After 5 min incubation at room temperature, the mixture was vortexed until it was completely homogenous. Centrifugation was then carried out at a speed of 8,000 rpm for 5 min. After that, centrifugation was run for 5 min at 8,000 rpm. After removing the visible supernatant, 350 µl of STE, 40 µl of 10% sodium dodecyl sulfate, and 10% ProtK were added. The solution was then homogenized for 2 h at 55°C using a rotating mixer. Subsequently, 40 µl of 5M NaCl, 400 µl of phenol solution, and 400 µl of chloroform isoamyl alcohol were added, and the mixture was homogenized for an hour at room temperature with a rotating mixer. After that, the mixture was centrifuged for 5 min at 12,000 rpm. Using a pipette, 400 µl of the generated supernatant was removed into a fresh 1.5 ml tube. Then, 800 µl of 100% ethanol (EtOH) was added, homogenized, and let stand at −20°C overnight. After centrifuging the mixture for 5 min at 12,000 rpm, the supernatant was removed. After adding 800 µl of 70% EtOH, the mixture was centrifuged for 5 min at 12,000 rpm. For 2 to 3 h, the DNA molecule precipitate at the tube’s bottom was dried. Once it was dry, 100 µl of TE buffer (80%) was added and homogenized, and the DNA was ready for further analysis or frozen and stored for long-term usage.

### Amplification of GH and IGF-1 gene

Amplification of GH and IGF-1 genes in Napu buffalo was carried out by polymerase chain reaction (PCR) technique using a thermal cycler machine. The annealing temperatures used for the GH and IGF-1 gene loci were 62°C and 68°C, respectively. The PCR solution used consisted of 2 µl DNA sample, 10 µl nuclease-free water, 0.5 µl forward primer, 0.5 µl reverse primer, and 12.5 µl MyTaq HS Red Mix (Bioline, UK). Primers used for PCR were designed using Oligo Explorer software (Table 1).

### Genotyping and visualization of DNA bands

Genotyping of GH and IGF-1 genes in Napu buffaloes using the polymerase chain reaction-restriction fragment length polymorphism (PCR-RFLP) technique using *AluI* and *SnaBI* enzymes. A total of 2 µl of PCR product was mixed with 1–2 units of restriction enzyme in 1x Buffer (Biolabs, New England) and then incubated at 37°C for at least 4 h. Visualization of genotyping bands was electrophoresed using a 2.5% agarose gel. A total of 5 µl of PCR-RFLP DNA was inserted into a 2.5% agarose gel and compared with a 100 bp marker. Genotyping was done based on the length of DNA fragments seen in Table 2.

### Sequencing

Direct Sanger sequencing analysis was carried out to see the similarities or mutations that occurred in the Napu buffalo samples after being compared with the comparator sequences from GenBank KC107770.1 for the GH gene and KC852883.1 for the IGF-1 gene.

**Table 1.** GH and IGF-1 gene primers.

No	Loci	Primer sequences	GenBank	Product length (bp)	Annealing temperature (°C)	Reference
1	GH	F:5'-GCT GCT CCT GAG GGC CCT TCG-3'	KC107770.1	342	62	Biswas et al. [17]
		R:5'-GCG GCG GCA CTT CAT GAC CCT-3'				
2	IGF-1	F:5-'ATT ACA AAG CTG CCT GCC CC-3'	KC852883.1	250	58	Ge et al. [18]
		R:5-'ACC TTA CCC GTA TGA AAG GAA TAT ACGT-3’				

**Table 2.** GH|*AluI* and IGF-1|*SnaBI* gene genotyping cut sites.

No	Loci	Genotype	Product length (bp)
1	GH	VV	236 bp
		LL	185 bp
		VL	236, 185 bp
2	IGF-1	AA	224, 26 bp
		BB	250 bp
		AB	250, 224, 26 bp

### Data analysis

Measurement data of Napu buffaloes were tabulated by age group and sex and analyzed descriptively. The strength of the association between variables was evaluated by computing correlation coefficients (r) between various morphometric measurements and BW. Multiple linear regression analysis was conducted using SPSS version 21 software (IBM, US) with the following equation: Y_ij_ = a + b_i_X_i_ + e_j_, where Y_ij_ is the dependent variable (BW), a is the constant, b_i_X_i_ is the independent variable, and e_j_ is an error.

Allele frequency is the ratio of the number of a particular allele to all alleles in a population. Allele frequency is calculated based on Nei and Kumar [16]. Genotype frequency is the ratio of the number of genotypes to the total population of genotypes. Genotype frequency is calculated based on:


$$x_{\mathrm{ii}}\;=\;\frac{\sum_{i=1}^{n}\mathrm{ni}}{N}$$

where *x_ii_* is genotype frequency ii, ni is the number of genotypes, and *N* is the total sample.

## Results

### BW and morphometric characteristics of Napu buffalo

The mean of BW and morphometric parameters of Napu buffaloes are presented in Table 3. The correlation coefficient between BW and morphometric parameters of Napu buffaloes is presented in Table 4. The correlation value of BW with the body size of Napu buffaloes has a positive value except for the correlation value of BW with the chest. The positive relationship from the correlation test results indicates that the higher the value of variable *X* (Napu buffalo body size), the higher the value of variable *Y* (BW). The results of regression analysis of morphometric parameters on the BW of Napu buffaloes are presented in Table 5.

### Polymorphisms of GH and IGF-1 genes of Napu buffalo

**Figure 2.**
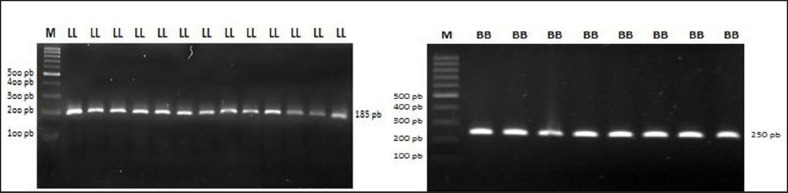
PCR-RFLP visualization results of GH|*AluI* and IGF-1|*SnaBI*.

**Figure 3.**
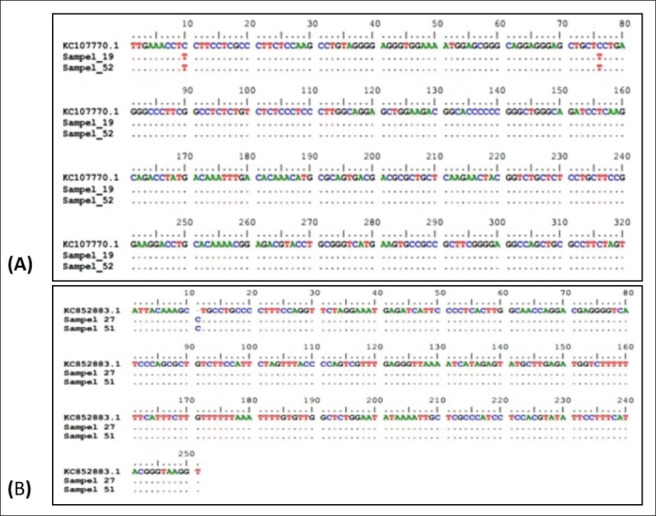
(A, B) PCR-RFLP results and sequencing results in Napu buffalo show monomorphic results.

PCR-RFLP visualization results of GH|*AluI* and IGF-1|*SnaBI* are shown in Figure 2. PCR-RFLP results show that the GH gene and IGF-1 gene in Napu buffalo are monomorphic, which is presented in Table 6. The genotype found in the Napu buffalo GH gene is 100% LL genotype, while the IGF-1 gene was found to be 100% BB genotype. The alleles found are 100% L alleles in the GH gene and 100% B alleles in the IGF-1 gene. Similar to the PCR-RFLP results, sequencing results in Napu buffalo showed monomorphic results (Fig. 3A and B).

## Discussion

### BW and morphometric characteristics of Napu buffalo

BW is an indicator of livestock productivity that can be assessed based on body measurements. According to Siamtiningrum et al. [4], livestock body measurements such as BL, height, and CG can be used to predict livestock productivity. In addition, Budianto et al. [19] stated that BW has a positive relationship with morphometric parameters in cattle.

The BW of female Napu buffaloes is influenced by age. The BW of female Napu buffaloes aged ≥1-< 2 years and ≥ 2-< 3 years was highly significantly different (*p *< 0.01). While the BW of male Napu buffaloes of different ages is not significantly different. In addition, Napu buffaloes with different sexes and the same age have BWs that are not significantly different.

Sex in Napu buffaloes aged ≥ 2-< 3 years affects the BL of Napu buffaloes highly significantly different (*p* < 0.01). In addition, age in female Napu buffaloes affects BL highly significantly different (*p* < 0.01). The results of the study by Nur et al. [20] state that female swamp buffaloes at different ages have significantly different BLs (*p *< 0.01). According to Pipiana et al. [21], varied environmental factors may affect buffalo BL.

Chest depth and CG in livestock are measures that indicate the dimensions of the rib cage. CG affects the body shape of livestock and is usually used as an indicator for estimating BW. Age and sex in Napu buffaloes were highly significantly different (*p *< 0.01) affecting the size of the chest and chest circumference. Males aged ≥1-<2 years and females aged ≥2-<3 years had highly significantly different CG (*p *< 0.01). In addition, females of different ages have chest measurements and CG that are highly significantly different (*p* < 0.01).

**Table 3.** Mean and standard error (SE) of BW and morphometrics parameters of Napu buffalo.

Age (Year)	Sex (*n*)	BW (kg)	BL (cm)	SH (cm)	CG (cm)	DD (cm)	HH (cm)
≥1-< 2	Male (5)	128.6 ± 5.97^Ad^	87.40 ± 3.01^A^	104.6 ± 1.44^ab^	135.2 ± 1.71^A^	51 ± 1.14^A^	106.4 ± 2.04^ac^
	Female (11)	118.1 ± 4.06^A^	90.45 ± 1.82^A^	99.64 ± 2.23^A^	135.18 ± 3.40^A^	50.36 ± 0.88^A^	105.2 ± 1.4^A^
≥ 2-< 3	Male (4)	175.7 ± 17.02^bd^	89.50 ± 0.50^A^	107.5 ± 2.5^b^	147.25 ± 4.77^AB^	54.5 ± 1.66^AB^	111.7 ± 1.65^bc^
	Female (19)	184.0 ± 9.59^B^	101.37 ± 1.25^B^	108.47 ± 1.72^B^	154.63 ± 3.64^B^	57.63 ± 1.34^B^	113.7 ± 1.31^B^

n: sample size, different superscripts in the same column indicate (*p < *0.05) significantly different and (*p < *0.01) highly significantly different (capital superscript).

**Table 4.** Correlation coefficient of BW with morphometric measures of Napu Buffalo for all age groups.

Variable	BW	Level of relationship
BW	1	
BL	0.649^**^	Strong
SH	0.727^**^	Strong
CG	0.586^**^	Moderate
DD	−0.028	Low
HH	0.842^**^	Very strong

** : Correlation coefficient between body variables shows a highly significant relationship (*p < *0.01).

HH in female Napu buffaloes of different ages has a high significant difference (*p *< 0.01). HH affects female buffalo reproduction. Female buffaloes with ideal HH may have ideal hip width, thus affecting the birthing process [20].

Age in female Napu buffaloes highly significantly (*p *< 0.01) influenced SH. Female Napu buffaloes aged ≥1-< 2 years and females aged ≥ 2-< 3 have significantly different SH sizes (*p *<0.01), while male Napu buffaloes at different ages are not different. This is by the research of Nur et al. [20], which found that age-influenced swamp buffalo are highly significantly different (*p *< 0.01).

Chest depth and CG in livestock are measures that indicate the dimensions of the rib cage. CG affects the body shape of livestock and is usually used as an indicator for estimating BW. Age and sex in Napu buffaloes highly significantly (*p *< 0.01) affected the size of the chest and CG. Males aged ≥1-<2 years and females aged ≥2-<3 years had highly significantly different CG (*p *< 0.01). In addition, females of different ages have chest measurements and CG that are highly significantly different (*p *< 0.01).

The significance value of the correlation test results of BW with BL, SH, CG, and HH is *p *< 0.01 with a correlation coefficient value of 0.5–0.8, which means that the variables have a moderate to very strong relationship.

According to Salsabela and Suhardi [22], the correlation coefficient value of 0.8–1 means that the two variables have a very strong relationship. The highest correlation coefficient value is BW and HH with a value of 0.842, which means it has a very strong relationship. This is in line with the research of Dahlan et al. [23], which states that the correlation value of HH and BW is 0.906, which is categorized as having a very strong relationship.

BW and BL as well as BW and SH have a correlation coefficient value of 0.649 and 0.727, respectively, which is categorized as having a strong relationship. This is by Dahlan et al. [23], who stated that BW has a positive relationship with the BL and SH of buffaloes. In addition, Soul et al. [24] stated that BL and SH have a significant positive relationship with BW in cattle.

BW and CG have a correlation coefficient of 0.586, which is categorized as having a moderate relationship. While the correlation coefficient of BW with CG has a value of −0.028. The negative relationship between BW and CG means that the higher the CG value of Napu buffaloes, the lower the BW value. Based on the correlation value (*R*) and determination value (*R*²), HH is the best variable in estimating Napu buffalo BW. This is indicated by the highest correlation value and determination value of 0.884 and 0.709, respectively.

Based on the results of regression analysis, HH can be used as a good predictor of BW. This is by the correlation value and determination value from the regression analysis of morphometric parameters of Napu buffaloes. The determination value of HH in Napu buffalo is 0.709, which means that 70.9% of Napu buffalo BW is influenced by HH with a significance level (*p *< 0.05) of 0.000.

Meanwhile, the regression equation between BW and body measurements of Napu buffaloes is BB= −398.768 + 0.836PB + 1.065TP + 0.687LD - 1.678DD + 3.225HH, with a coefficient of determination (*R*²) of 0.851, which means that 85.1% of Napu buffalo BW is influenced by BL, SH, chest circumference, chest depth, and HH, while the rest is influenced by other factors. The equation can be used as an estimation of Napu buffalo BW.

**Table 5.** Regression equation of morphometrics on BW of Napu buffalo.

No	Parameters	Regression Equation	Determination value (*R*^2^)	Significance(*p <*0.05)
1	BL	BW = −159.19 + 3.324BL	0.421	0.000
2	SH	BW = −256.235 + 3.926SH	0.528	0.000
3	CG	BW = −39.920 + 1.353CG	0.343	0.000
4	DD	BW = 163.224 − 0.104DD	0.001	0.865
5	HH	BW = −449.578 + 5.510HH	0.709	0.000
6	BL + SH	BW = −323.978 + 1.872BL + 2.876HH	0.624	0.000
7	BL + CG	BW = −179.910 + 2.394BL + 0.750CG	0.494	0.000
8	BL + DD	BW = −142.283 + 3.526BL − 0.664DD	0.451	0.000
9	BL + HH	BW = −455.233 + 0.974BL + 4.720HH	0.730	0.000
10	SH + CG	BW = −257.719 + 3.152SH + 0.570CG	0.568	0.000
11	SH + DD	BW = −254.751 + 4.519SH − 1.176DD	0.617	0.000
12	SH + HH	BW = −449.949 − 0.037SH + 5.549HH	0.709	0.000
13	CG + DD	BW = −37.432 + 2.115CG − 2.087DD	0.552	0.000
14	CG + HH	BW = −452.047 − 0.033CG + 5.577 HH	0.709	0.000
15	DD + HH	BW = −453.591 − 1.133DD + 6.106HH	0.794	0.000
16	BL + SH + CG + DD + HH	BW = −398.768 + 0.836BL + 1.065SH + 0.687CG − 1.678DD + 3.225HH	0.851	0.000

**Table 6.** Genotype frequency and allele frequency of GH|Alul and IGF-1|*SnaBI* genes of Napu buffalo.

Loci	Genotype (%)			Allele (%)	
GH|*Alul*	LL (185)	LV (236, 185)	VV (236)	L	V
	100	0	0	100	0
IGF-1|*SnaBI*	AA (224, 26)	AB (150, 224, 26)	BB (250)	A	B
	0	0	100	0	100

### Polymorphisms of GH and IGF-1 genes of Napu buffalo

In genetic evolution research, the degree of genetic variety within a population can serve as a measure and distinguisher of particular characteristics. The allele frequency indicates the degree of genetic variety within a population. The ratio of one allele to the total alleles in a population is called allele frequency. Based on molecular markers GH|*AluI* and IGF-1|*SnaBI* Napu buffaloes have very low diversity. This can be seen from one genotype that has a genotype frequency and allele frequency of 100%. This can be caused by the inbreeding process. Nei and Kumar [16] stated that a high level of inbreeding can reduce the diversity of a population.

The GH gene is one of the genes that influence growth traits in buffalo. The GH gene encodes GH, which has many functions. GH is required in the process of tissue growth, fat metabolism, and normal growth. In addition, GH has a role in reproductive capabilities such as ovulation rate, fertility, and embryo quality [25]. Several previous studies related to GH gene diversity in buffaloes had similar results. The Napu buffalo research findings are consistent with those of Andreas et al. [26], who discovered that in five Indonesian buffalo populations (Siborong-boring-Medan, Lebak-Banten, Pandeglang-Banten, Semarang-Central Java, and Mataram-NTB), the GH|Alul gene at the 432 pb loci (intron 4, exons 4-5) is monomorphic and has 2 genotypes.

In contrast to the research of Unal et al. [27], who found 8 SNPs of the GH|Alul gene in buffaloes in Turkey along 404 pb (intron 4, exon 5, and 3’UTR) which resulted in 4 genotypes and changed the amino acid lysine to arginine. Konca and Akyuza [28] also found the GH|*Alu*l exon 5 gene loci in Anotalia water buffalo is polymorphic with three genotypes, namely LL, LV, and VV genotypes. The highest genotype frequency was LL (0.755), and the lowest genotype frequency was VV (0.017).

IGF-1 is a major growth signaling member [29]. The IGF-1 gene has various roles in physiological and metabolic functions, where IGF-1 and GH are involved in the sumatropic axis. IGF-1 is a mediator of many biological processes, such as glucose uptake, stimulation of myogenesis, and interest in DNA, protein, RNA synthesis, and cell proliferation [30]. In addition, IGF-1 also plays a role in muscle growth and development.

The results indicated that the IGF-1 gene in Napu buffalo is monomorphic. The genotype found in Napu buffalo is the 100% BB genotype. This is similar to the research of Margawati et al. [31], which found that the IGF-1 gene in swamp buffalo and river buffalo is monomorphic.

The present study shows that body size and weight in Napu buffaloes may be affected by age and sex. The GH and IGF-1 genes in Napu buffaloes exhibited monomorphism. The results may be attributable to the limited sample size studied at this time. Future studies should involve a bigger sample size with comparators.

## Conclusion

Age and sex in Napu buffaloes may affect BW and body size. The GH|*Alul* and IGF-1|*SnaBI* loci in Napu buffalo are monomorphic. The genotypes found at the GH|*Alul* and IGF-1|*SnaBI* loci are LL and BB. The results of this study are very surprising compared to other animal studies. The results of the study can be the basis for further research on the same population or different populations. We can carry out further research by increasing the number of Napu buffalo samples and comparing them with existing buffalo populations in Indonesia.

## List of abbreviations

BL, body length; BW, body weight; CG, chest girth; DD, chest depth; DNA, deoxyribonucleic acid; EtOH, ethanol; GH, growth hormone; HH, hip depth; IGF-1, insulin-like growth factor-1; NaCl, sodium chloride; PCR, polymerase chain reaction; PCR-RFLP, polymerase chain reaction-restriction fragment length polymorphism; ProtK, protein kinase; rpm, revolution per minute; SH, shoulder height; SNPs, single nucleotide polymorphism; TE, tris-EDTA.
